# Waugh's Syndrome in an Adult Patient: A Case Report and a Review of the Literature

**DOI:** 10.7759/cureus.51786

**Published:** 2024-01-07

**Authors:** Khaled Afasha, Nada Alabdulwahid, Atif Dawood

**Affiliations:** 1 General Surgery, King Abdulaziz Hospital and Oncology Center, Jeddah, SAU

**Keywords:** waugh's syndrome, intestinal obstruction, malrotation, intussusception, cystic fibrosis

## Abstract

Waugh's syndrome is a rare disease that typically presents as acute-onset intussusception caused by gut malrotation. This disease, while generally reported during infancy or childhood, can also affect adults. This case report describes the rare case of Waugh's syndrome in an adult with cystic fibrosis. The patient, a 19-year-old male, was admitted one week after the onset of pain and distention in the abdomen, with vomiting alternating with constipation. He had visited emergency rooms in the past and was taking symptomatic treatment, but on arrival at our facility, he developed a tonic-colonic convulsion. The evaluation showed respiratory acidosis, severe hyponatremia, and abdominal distension with an empty rectum. The patient was discovered to have colo-colonic intussusception by imaging, and he was admitted into the intensive care unit before exploratory surgery. Surgical findings showed intestinal malrotation with the cecum lying in the left upper quadrant and ilio-colic intussusception extending to the mid-rectum. A distal ileal resection was required for partial reduction, leaving a residual segment needing an ileostomy. Histopathology performed after surgery found a blocked lumen, edema hemorrhage, and necrosis. Following discharge, the patient's recovery was hampered by a high-volume stoma. He had to be readmitted for electrolyte correction and hydration as well as preparation of the normal passage of foods through his system. Later follow-up showed that the output of the stoma had been brought under control, and nutritional status greatly improved. The patient planned to have a stoma reversal after three months. This case emphasizes the need for a multidisciplinary approach to treating Waugh's syndrome in adult patients.

## Introduction

Waugh's syndrome is a very rare and complex medical condition that includes various gastrointestinal abnormalities. It can affect the digestive system, resulting in an array of symptoms that require prompt management [1]. It is the association between intestinal malrotation and intussusceptions. Commonly, pediatric surgeons are familiar with intussusception, but general surgeons seldom see it. Adult intussusception is rare, accounting for less than 5% of all intestinal obstructions. Of these, half are associated with neoplasms. This disease usually represents an incidental finding with unrelated disease on image studies, laparotomy, or even autopsy [2]. The principal complications of intestinal malrotation are obstruction due to midgut volvulus, internal hernia, or adhesion band. In fact, to our knowledge, there has been only one prospective study published on Waugh's syndrome. Out of the 49 cases, 18 cases of Waugh's syndrome involved intussusception, a condition in which increased mobility due to a slackened bowel wall [3]. Some authors maintain that Waugh's syndrome may be surprisingly common and not recognized in intussusceptions, which have been treated non-operatively. Many other physicians believe it is much more frequent but undiscovered since reduction by enema suffices for many patients with intussusception, and operative exploration doesn't take place [4]. Most of the information on Waugh's syndrome is derived from the pediatric population as more pediatric cases have been published compared to adults. In this case, we discuss a rare presentation of Waugh's syndrome in adult patients.

## Case presentation

A 19-year-old male with a known history of cystic fibrosis presented to our emergency room (ER) in a post-ictal state. The patient had a week-long history of abdominal pain, distention, vomiting, and constipation. Despite seeking medical advice twice in another hospital's ER and receiving stool softeners and analgesics, his symptoms persisted. Upon presentation to our ER, the patient experienced a tonic-clonic convulsion, followed by confusion, tachycardia, hypotension, cachexia, and abdominal distension with an empty rectum per rectal examination. Arterial blood gases revealed respiratory acidosis and laboratory tests showed severe hyponatremia (sodium levels: 121 mmol/L; normal range: 136-145 mmol/L). Other parameters on presentation included a white blood cell count (WBC) of 7.2 K/u, a hemoglobin (HGB) level of 11.8 g/dL, and an albumin level of 33.2 g/L. 

**Figure 1:**
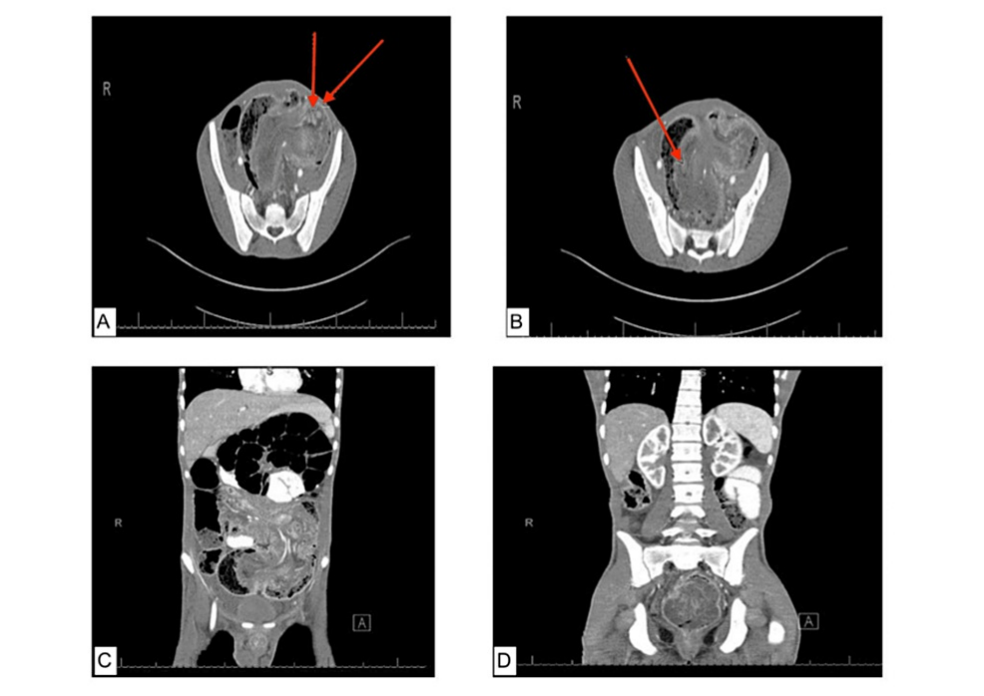
Computer tomography (CT) imaging A, B) Axial cuts of CT abdomen-pelvis with triple contrast (IV-oral-rectal) and the arrow pointing to the area of colo-colonic intussusception. C, D) Coronal cuts of CT abdomen-pelvis with triple contrast (IV-oral-rectal) with the intussusceptum reaching down to the rectum with mild subsequent small bowel dilatation.

The patient was promptly resuscitated, intubated in the intensive care unit (ICU), and underwent hyponatremia management. At this stage, the surgical team was involved in the evaluation. After stabilization, computer tomography (CT) of the head and abdomen was done, which revealed a colo-colonic intussusception reaching the rectum with subsequent dilatation of small bowel and minimal pelvic abdominal free fluid (Figure 1)

**Figure 2:**
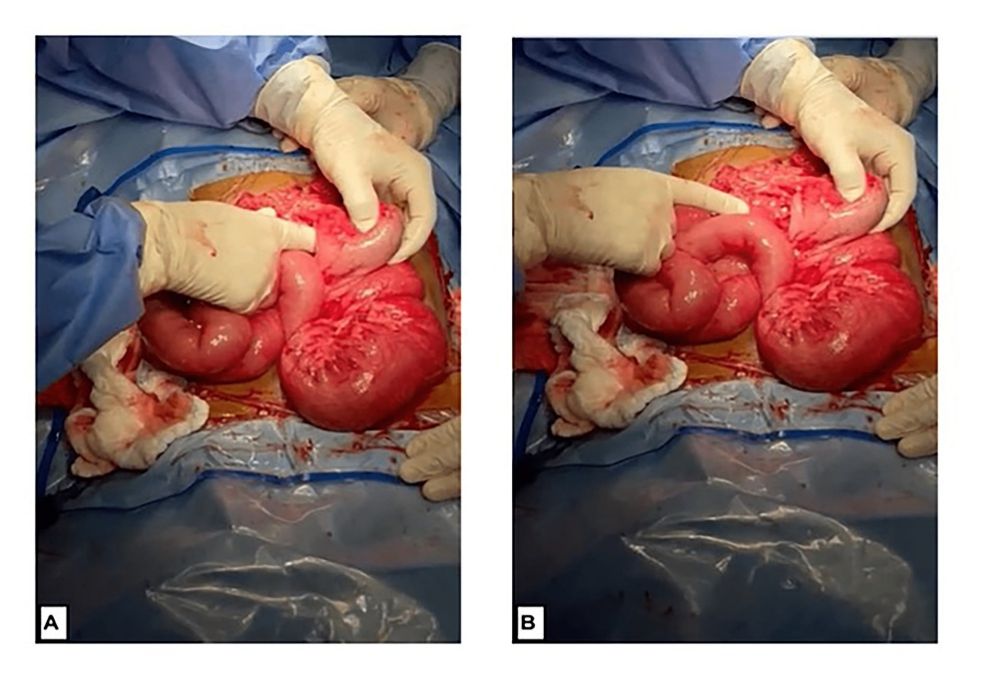
Intraoperative imaging A, B) Intraoperative finding of intestinal malrotation whereas the cecum found in the left upper quadrant with ilio-colic intussusception

The patient was admitted to the ICU and received resuscitation, monitoring, correction of acidosis, and decompression. Subsequently, the patient underwent exploratory surgery in the operative room (OR), revealing intestinal malrotation. The cecum was located in the left upper quadrant, accompanied by ilio-colic intussusception. The ileum extended to the mid-rectum, exhibiting diffuse small bowel dilation. Attempts were made to manually reduce the intussusception, resulting in partial success. Consequently, a decision was made to perform a resection of the distal ileum up to the upper rectum. Despite using a gastrointestinal anastomosis (GIA) stapler on the upper rectum, a portion of the ileum remained in the distal rectum, necessitating the creation of an ileostomy (Figure 2).

Post-operatively, the histopathology report showed a prolapsing bowel inside of the large bowel along with feces, which exhibited semi-complete luminal obstruction. The bowel wall displayed edema, hemorrhage, and necrotic areas, with no discernible masses or solid regions. Following surgery, the patient remained intubated and sedated in the ICU to monitor pulmonary function due to his cystic fibrosis and to control hyponatremia and any newly developed convulsion. The patient's recovery in the hospital was uneventful, and he was discharged on day seven in good condition after successfully tolerating oral intake and a functional stoma. However, three days after discharge, the patient returned to the ER due to a high-output stoma, dehydration, and electrolyte imbalances leading to hyponatremia. Consequently, the patient was readmitted for rehydration, electrolyte correction, close monitoring of fluid intake and output, and management of stoma output using loperamide. The dose of pancreatic enzymes was increased, and a dietitian was consulted to address chronic constipation, underweight concerns, and nutritional needs specific to cystic fibrosis, including sodium replacement. After a few days, the stoma output was successfully controlled, and the patient was discharged with plans for close follow-up. Subsequent follow-up appointments indicated the patient's positive progress, including increased oral intake, weight gain, and adherence to medications and instructions. The patient expressed a desire to undergo stoma reversal after completing three months post-operation.

## Discussion

Waugh's syndrome is a rare syndrome that has been mostly reported in infants; however, few cases have also been reported in adults. So far, less than a hundred cases of Waugh's syndrome have been reported in children [5]. This syndrome was first described by Waugh in 1911 [6]. Brereton et al. conducted a prospective study where they identified intestinal malrotation-associated intussusceptions in 15 patients, coining the term "Waugh's syndrome" after George E. Waugh [7]. Although intussusception causes only 5% of cases of intestinal obstruction in adults, about 80 to 90% are secondary to underlying pathologies [8]. Adults, unlike children and infants present with intussusception, generally have a lead point. The leading points are vascular anomalies, tumors, foreign bodies, lymphoid hyperplasia, suture lines, Meckel diverticulum, inflammatory lesions, and postoperative adhesions [2]. 

Malignant or benign neoplasms account for around 65% of the adult intussusceptions [8]. A moveable right colon by a long meso-colon results from an intestinal malrotation. It is assumed that a mobile cecum may result in prolapse of the ileocecal valve into the cecum, plus the outcome is intussusception. The finding of this double pathology is made intraoperatively. The preoperative diagnosis of this syndrome is nearly impossible. Ultrasonography and abdominal radiographs are the initial aids to a careful medical inspection [9]. In this case, a CT scan was performed that showed a colo-colonic intussusception with intussusceptum reaching the rectum with subsequent dilatation of small bowel and minimal pelvic abdominal free fluid. We found that there was intestinal malrotation, whereas the cecum was found in the left upper quadrant with ileocolic intussusception, and the ileum reached up to the mid of the rectum with diffuse dilation of the small bowel. In the current case study, only partial reduction of the ileum was obtained manually, so it was decided to do a resection of the distal ileum up to the upper rectum. The optimum management of adult intussusception is quite a debatable matter. The majority of the authors suggested that surgery is essential, but it is a management of choice in adults. There is a disagreement over whether the intussusception ought to be reduced before resection or if it must be avoided because of an augmented risk of contamination and perforation [8]. 

Effective endoscopic resection has also been described for benign lesions that cause intussusception, but it must be dealt with with cautiousness owing to high complication risks. Expert hands can also perform minimally intrusive surgery for resection of the targeted fragment, and this will decrease the hospital stay and pain of the patients [10]. In the present case, resection of the distal ileum up to the upper rectum was done, but part of the ileum was retrieved from the distal rectum, and an ileostomy was performed. For intestinal malrotation, a Ladd procedure is necessary, including dividing the Ladd band, mobilizing the duodenum in addition to the right colon, adhesiolysis nearby mesenteric vessels, and appendectomy [11]. The patient in the present case had a medical history of cystic fibrosis and was suffering from abdominal pain for the last seven days. Hypernatremia was also being managed side by side with resuscitation. Surgery was performed, and the patient was followed up for seven days and then discharged. Positive outcomes were seen on a long-term follow-up. 

There is a paucity of literature on Waugh syndrome in adults. To identify relevant literature on the topic, a systemic search was carried out in PubMed and Google Scholar. A combination of "Waugh's syndrome" and "adults" keywords was used during the search. A total of five case reports were identified in adults. The available literature is presented below in the form of a descriptive table (Table 1). 

**Table 1:** Description of the available literature of Waugh's syndrome in adults GIST - gastrointestinal stromal tumor

Author and Year	Age	Gender	Procedure	Operative findings	Outcome
Hsieh et al. (2008) [2]	56	Male	Conservative treatment and later managed with laparotomy. The ladd procedure was also applied.	Spindle cell neoplasm, CD34, and SMA were revealed in an immunohistochemical profile, and GIST: gastrointestinal stromal tumor markers; PKC (-) and CD117 (-). The discoveries were similar to a provocative fibroid polyp.	The postoperative outcomes were uneventful.
Chaudhary et al. (2012) [12]	25	Female	Laparotomy and surgery	Ileocolic intussusceptions reaching up to the middle of the ascending colon, duodenojejunal flexure was lying onto the right of the middle line (abnormal position). Changes in distal small bowel.	The patient was discharged after 6 days and postoperative outcomes were uneventful.
Gandhi et al. (2019) [8]	60	Female	Colonoscopy and surgery	The small bowel loops and duodenojejunal flexure are present on the right side. The ascending colon, hepatic flexure, and caecum were unsettled and were present in the mille line. Colonic intussusception was found with a leading point (palpable large polyp).	She was discharged after 6 days of surgery, and her postoperative period was uneventful.
Sausa et al. (2020) [13]	56	Female	Right hemicolectomy	Sall bowel restriction to the right side. Waugh syndrome was diagnosed and no organic lesions were recognized upon imaging, analysis of the specimen, and during the surgery.	The patient was not followed up after surgery in the reported case.
Induchoodan et al. (2021) [9]	26	Female	Laparotomy	Upon laparotomy, a long mesocolon of the hepatic flexure and ascending colon was found. The duodenojejunal flexure is present lateral to the ileocaecal junction, and a broad peritoneum band was found as of the ascending colon to the crosswise facet of the duodenum. The terminating part of the ileum was invaginating into the insecurely lying inferiorly half of the ascending colon.	A manual reduction attempt failed and there was a need to perform a limited right hemicolectomy.

## Conclusions

In conclusion, this case demonstrates the complexity and difficulty of gastrointestinal complications in cystic fibrosis patients. The case of a 19-year-old male with cystic fibrosis and colo-colonic intussusception, which developed into bowel prolapse, demonstrated the importance of rapid assessment and treatment. Maybe the underlying intestinal malrotation was overlooked, and this contributed to continuing symptoms even after medical visits. This case was handled effectively through an interdisciplinary approach by combining critical care with the talents of surgery and specialized cystic fibrosis therapy. After stabilizing temporarily in an intensive-care unit, more detailed analysis through imaging studies and exploratory surgery finally enabled a clear-cut anomaly of structure to be seen. These included a resection of the distal ileum and an ileostomy creation to deal with the extensive infiltration by the bowel, as well as postoperative fecal impaction. After surgery, the patient had problems with a high-output stoma. Not only does this demonstrate that postoperative care is difficult, but treatment for cystic fibrosis also involves many different complications.
